# AIS-Informed and Land-Aware Eco-Routing for Maritime Decision Support Using Machine Learning and Metocean Data

**DOI:** 10.3390/s26196092

**Published:** 2026-09-25

**Authors:** Nikolaos Douros, Dimitrios Kaklis, Ioannis Kontopoulos, Konstantinos Tserpes, Iraklis Varlamis

**Affiliations:** 1 Department of Informatics and Telematics, Harokopio University of Athens, 176 76 Athens, Greece; it2022127@hua.gr; 2 Danaos Research Center, 185 45 Piraeus, Greece; kaklis1992@gmail.com; 3 Archimedes Unit, Athena Research Center, 157 80 Athens, Greece; 4 School of Electrical and Computer Engineering, National Technical University of Athens, 157 80 Athens, Greece; ikontopoulos@mail.ntua.gr (I.K.); tserpes@mail.ntua.gr (K.T.)

**Keywords:** AIS, maritime routing, eco-routing, geospatial data analytics, land avoidance, metocean data, fuel-consumption prediction, decision support

## Abstract

Geospatial sensing and data analytics can support maritime risk and safety management by transforming vessel-movement and environmental observations into transparent evidence for route assessment. Within this context, reducing maritime fuel consumption requires routing methods that exploit historical vessel-movement data while representing geographical and metocean conditions explicitly. This study contributes an AIS-informed, land-aware eco-routing framework that integrates geospatial data processing, machine-learning-based fuel estimation, and weather-screening analysis to generate minimum-predicted-fuel routes and examine their exposure to environmental conditions. Minimum-predicted-fuel routing is formulated as a shortest-path problem and solved using Dijkstra’s algorithm. The search operates on an undirected graph constructed from historical AIS-derived segments and augmented with short stitching links screened using a global land mask. Edge costs are estimated by a Random Forest fuel-consumption model using vessel speed and draft together with current wave height and wind speed. The framework is evaluated through a paired comparison with a historical AIS observation sequence and a controlled nine-combination factorial weather-screening experiment on two routes using frozen execution-time weather snapshots. In the paired comparison, the generated route had 25.54% lower model-estimated fuel consumption and 11.65% shorter reconstructed travel time, but 9.31% greater distance and higher distance-weighted mean wave height and wind speed. This baseline difference coincided with higher assigned generated-route speeds and was sensitive to the fuel-model training-speed domain, so it is treated as an illustrative result. Weather screening either disconnected the graph or left the fuel-minimising path unchanged. It also left every feasible path on the shorter route unchanged. These findings illustrate how geospatial sensing and data analytics can expose interactions among fuel efficiency, metocean exposure, graph connectivity, and missing-weather assumptions, while supporting transparent maritime risk and safety decision-making. By combining AIS sensor observations, weather data, and historical vessel paths, the proposed approach identifies fuel-optimal candidate routes within a geographically and environmentally screened network and provides evidence for safety-oriented route assessment.

## 1. Introduction

Global maritime transport remains the indisputable backbone of international trade, handling about 80% of the volume of international trade in goods [1]. However, the heavy reliance on fossil fuels, particularly Heavy Fuel Oil (HFO), makes the shipping industry a significant contributor to global greenhouse gas (GHG) emissions. In response to the escalating climate crisis, the International Maritime Organization (IMO) has introduced highly stringent regulatory frameworks. Key amongst these are the Energy Efficiency Existing Ship Index (EEXI) and the Carbon Intensity Indicator (CII), which mandate a progressive, quantifiable reduction in the carbon footprint of existing fleets.

For shipping companies, fuel consumption constitutes the vast majority of operational expenditures (OPEXs), heavily influencing overall profitability in a highly competitive market. Consequently, optimising vessel trajectories—commonly referred to as weather routing or eco-routing—has emerged as the most immediate, cost-effective, and practical solution. Unlike the adoption of alternative green fuels (such as ammonia, methanol, or hydrogen), which require massive infrastructural investments, supply chain overhauls, and expensive engine retrofitting, digital voyage optimisation can be deployed immediately across existing fleets.

Maritime routing is also a geospatial sensing and safety-management problem. AIS transponders automatically exchange vessel identity, position, course, speed, navigational status, and other safety-related information with ships and shore stations, providing a distributed source of maritime-traffic observations under the IMO’s SOLAS framework [2]. These observations improve situational awareness and support traffic monitoring, but their safety value depends on data reliability, correct human interpretation, and integration with other navigational information; early work by Harati-Mokhtari et al. established the importance of AIS data quality and human factors in safe navigation [3]. At the analytical level, historical AIS streams can be transformed into learned waypoints, traffic patterns, route predictions, and anomaly indicators, as demonstrated by the foundational trajectory-knowledge framework of Pallotta et al. [4]. When combined with spatial land information and metocean observations, these sensor-derived patterns can provide a richer basis for assessing candidate routes than distance or fuel cost alone.

Several challenges nevertheless limit the direct translation of maritime sensor data into operationally safe routes. AIS records may contain missing observations, irregular sampling, erroneous manually entered attributes, reception gaps, or behaviour that is not representative of future conditions. Weather observations and forecasts introduce additional differences in spatial resolution, update time, uncertainty, and coverage. The resulting heterogeneous data must be aligned spatially and temporally, processed at graph scale, and incorporated into an optimisation problem that balances fuel, time, environmental exposure, and connectivity without treating historical traffic as proof of navigational safety. More generally, AIS-based intelligent navigation spans route extraction, trajectory prediction, collision analysis, anomaly detection, and path planning, each with distinct data-quality and modelling requirements [5]. Authoritative charts, bathymetry, regulatory constraints, vessel-response limits, and expert validation therefore remain necessary for operational use.

This paper introduces a data-driven eco-routing framework that combines historical Automatic Identification System (AIS) data, Geographic Information Systems (GIS), current metocean API data, and Machine Learning (ML). It uses AIS-derived navigational segments to constrain the search space and screens newly created graph links against land. The resulting route is a fuel-efficiency decision-support output within the modelled graph; navigational planning and operational safety decisions remain the responsibility of qualified operators.

The individual tools used here are not presented as new algorithms: Random Forest regression, Dijkstra shortest paths, AIS-derived navigation graphs, spatial indexing, and caching are established components. The contribution is the specific prototype design that combines an inherited AIS-network construction idea [6], a published fuel-rate model family [7], execution-time metocean assignment, land-screened graph stitching, and weather-threshold screening into one reproducible route-assessment workflow. This differs from conventional weather-routing studies that optimise over forecast grids or dynamic-programming/isochrone formulations [8,9,10,11], and from AIS-analytics studies that mainly extract or predict traffic patterns without evaluating route-level fuel and metocean exposure.

The main contributions of this work are as follows:

- an AIS-informed geospatial graph that preserves evidence of historical vessel movement and augments local connectivity through land-screened stitching links;
- an edge-cost model that combines vessel speed and draft with current wave and wind observations to estimate fuel consumption, enabling a minimum-predicted-fuel shortest-path search;
- a paired comparison of the generated path with a historical AIS observation sequence using common fuel, distance, reconstructed-time, and metocean-exposure descriptors as an illustrative evaluation of the framework output, not as an independent validation experiment; and
- a controlled factorial weather-screening analysis that quantifies edge exclusions, route connectivity, path changes, and sensitivity to missing-weather treatment.

The remainder of the paper is organised as follows. Section 2 reviews fuel-consumption modelling, voyage optimisation, AIS analytics, and metocean-aware routing. Section 3 presents the proposed methodology, including graph construction, spatial discretisation, land screening, environmental inputs, fuel estimation, and shortest-path optimisation. Section 4 describes the system implementation and its scalability mechanisms, including the modular architecture, spatial querying, graph stitching, caching, and downsampling. Section 5 reports and discusses the AIS comparison and weather-screening experiments. Finally, Section 6 summarises the findings, limitations, and directions for future work.

## 2. Background and Related Work

### 2.1. Fuel Oil Consumption Estimation: From Physics-Based to Data-Driven Models

The estimation of ship fuel consumption has traditionally relied on semi-empirical hydrodynamic and naval architecture models [12]. Methods such as the Holtrop–Mennen approach compute the total resistance of a ship as the sum of various components (frictional resistance, wave-making resistance, appendage resistance, etc.). While theoretically sound in controlled, calm-water environments or towing tank experiments, these “white-box” models exhibit significant limitations in real-world applications. They often fail to accurately account for nonlinear operational degradation over time, such as hull biofouling, propeller roughness, and age-related engine wear. Furthermore, accurately modelling added resistance in irregular, multi-directional sea states remains highly complex.

Recent literature has heavily pivoted towards data-driven approaches. Kaklis et al. [13] demonstrated that ML models, particularly when trained on continuous, high-frequency onboard sensor telemetry, can accurately predict Fuel Oil Consumption (FOC). These “black-box” or hybrid models capture complex vessel-specific nonlinearities and intricate environmental interactions that pure physics-based models miss, dynamically adapting to the vessel’s actual operational state.

### 2.2. Voyage Optimisation and Spatial Constraints

In the domain of voyage optimisation, algorithms must navigate complex, continuous ocean spaces while strictly avoiding landmasses, shallow waters, and treacherous weather systems [8]. A complete review of weather-routing algorithms is outside the scope of this methodological paper; comprehensive taxonomies and recent reviews are provided by Zis et al. [8], Walther et al. [9], and Chen et al. [11]. Traditional routing often relies on the Isochrone method or dynamic programming. Representative implementations include operational weather-routing systems [14,15], three-dimensional dynamic-programming formulations [10,16], and graph- or hybrid-optimisation approaches for multi-objective voyage planning [17,18]. However, applying these algorithms directly to vast oceanic grids leads to the curse of dimensionality and unacceptable computational overheads. The present paper does not provide a controlled runtime comparison with these traditional solvers; it focuses on an AIS-informed graph implementation and reports its algorithmic design choices.

A major challenge in autonomous, graph-based route generation is “Data Scarcity” in remote ocean areas. If nodes and edges are generated blindly via mathematical grids, the algorithm might attempt to traverse unnavigable straits or shallow reefs. To overcome this, Varlamis et al. [6] proposed an innovative methodology for extracting robust navigation networks by clustering multi-vessel trajectory data (Figure 1). Our research builds directly upon this foundation, utilising pre-computed historical motion polygons to constrain the search space of the graph.

### 2.3. AIS, Safety, and Metocean Routing

AIS trajectories provide an empirical record of vessel movement and can reveal commonly used corridors, speeds, headings, and traffic patterns. Their value for safety assessment, however, depends on the model layered on top of the trajectories. For example, collision-risk studies use encounter geometry and measures such as closest point of approach and time to closest point of approach, together with traffic and vessel characteristics, to quantify interactions between vessels [19]. An AIS track or an AIS-derived graph segment alone therefore provides evidence of prior movement, not a direct measure of collision risk, charted-hazard clearance, or compliance with navigational rules.

Metocean-aware routing has a similarly broader scope than fuel prediction alone. Established weather-routing approaches evaluate forecast wind, waves, vessel response, and operational constraints to seek acceptable trade-offs among voyage time, fuel use, added resistance, and exposure to adverse conditions [8,10,11]. In safety-oriented formulations, navigation risk is represented explicitly as a vessel- and metocean-dependent optimisation objective, rather than inferred solely from the fuel cost of a route [20].

These studies motivate the use of AIS and metocean information in the present decision-support framework, while defining its boundary. The baseline prototype uses historical AIS-derived segments to constrain its candidate graph and current wave height and wind speed as inputs to edge-level fuel prediction. A separate experimental mode removes edges above specified wave and wind limits before repeating the same fuel-minimisation search. It does not calculate encounter-based collision risk, vessel operability, or a validated metocean safety index. Accordingly, its outputs should be interpreted as geographically constrained fuel-minimising and weather-screened route suggestions for qualified operators to assess with authoritative charts, forecasts, traffic information, and vessel-specific operating guidance.

## 3. Proposed Methodology

The proposed methodology transforms historical vessel movements and current metocean observations into a weighted navigation graph and searches this graph for a minimum-predicted-fuel route. The process comprises spatial graph construction and land screening, assignment of metocean inputs, edge-level fuel estimation, and shortest-path optimisation.

### 3.1. Spatial Discretisation and Land Avoidance

The continuous maritime space is discretised into a graph G=(V,E), where *V* represents geographic waypoints and *E* represents maritime segments. The current prototype uses an undirected graph: the same stored edge can be traversed in either direction and has one symmetric set of distance, speed, weather, and fuel-cost attributes. This is a static simplification suitable for the present proof-of-concept, but it does not model direction-dependent currents, traffic lanes, reverse-heading manoeuvres, or time-varying conditions. Repeated observations contribute to the endpoint speed averages used during edge construction; if the same unordered edge is inserted more than once, the NetworkX graph stores one edge for that node pair with the attributes assigned by the latest insertion. To reduce the risk that newly created graph links cross land, a land-mask screen is applied during the graph-stitching phase.

The route graph contains AIS-derived segments and additional local stitching links that connect nearby graph nodes. For a proposed stitching link of at least 2 NM, ten uniformly spaced samples are generated along the straight line joining its endpoints. The two samples closest to each endpoint are omitted to reduce false rejections around ports and coastlines; the remaining six interior samples are queried against the global_land_mask land classifier [21]. The link is rejected if any retained sample is classified as land. The validity condition for such a stitching edge is as follows:

(1) $$e_{ij}\;\mathrm{is}\;\mathrm{valid}\Leftrightarrow \forall K_{s}\in S_{ij}:L\left(K_{s}\right)=0,$$

where $e_{ij}$ denotes the candidate stitching edge between graph nodes *i* and *j*, $S_{ij}$ is the retained set of sampled interior points along that edge, and $L(K_{s})$ is the global_land_mask classifier output for sample $K_{s}$, with 1 denoting land and 0 denoting water. The edge is therefore marked as valid only when all retained samples are classified as water. Original AIS-derived segments enter the graph as historical observations and are not independently re-screened by this procedure. This is a coarse geographical feasibility check that does not represent bathymetry, navigational hazards, traffic-separation schemes, restricted areas, or vessel-specific under-keel-clearance requirements. For retained edges, the great-circle distance is calculated using the Haversine formula:

(2) $$d_{ij}=2R\arcsin\left[\sqrt{\sin^{2}\left(\frac{\phi_{j}-\phi_{i}}{2}\right)+\cos\phi_{i}\cos\phi_{j}\sin^{2}\left(\frac{\lambda_{j}-\lambda_{i}}{2}\right)}\right],$$

where *R* is the Earth’s radius, $\phi_{i},\phi_{j}$ are endpoint latitudes, and $\lambda_{i},\lambda_{j}$ are endpoint longitudes. Figure 2 illustrates this land-screening step by showing how candidate stitching links are accepted or rejected according to the sampled land-mask checks.

### 3.2. Metocean Inputs for Fuel Prediction

Each graph edge is associated with a feature vector that combines vessel operating characteristics with representative metocean conditions. Speed over ground and mean draft describe the vessel state, while significant wave height and wind speed characterise environmental exposure along the edge. Thus, the cost of crossing an edge is not taken from the historical AIS voyage as an observed fuel value; it is estimated from the edge speed and draft together with the weather conditions assigned at evaluation time. These variables are supplied jointly to the fuel-consumption model, thereby allowing metocean conditions to influence the routing objective through the predicted edge cost. In this experiment, both the original route in the AIS dataset and the algorithm-generated route are evaluated with weather retrieved at the time of execution, rather than with a time-matched historical hindcast. The results should therefore be interpreted as a snapshot-based route assessment rather than as a hindcast validation of the 2016 AIS voyage. In the baseline formulation, environmental variables do not constitute independent risk penalties. An optional screening stage can instead impose admissibility constraints by removing edges whose conditions exceed prescribed limits before shortest-path optimisation. A common environmental field is retained across scenarios so that observed differences can be attributed to the screening limits rather than to changing input conditions.

Speed is an assigned edge attribute, not an optimisation decision variable. For AIS-derived graph edges, node speeds are averaged from positive historical segment speeds observed at the two endpoint nodes; the edge speed is then the mean of the two endpoint averages. Newly created stitching links use the same endpoint-average rule, so they do not introduce an independently optimised speed. In the paired comparison, the AIS reference route uses the recorded AIS speed for each retained segment, while the generated route uses the speed stored on each selected graph edge. Both alternatives use the same scalar mean-draft input retrieved from the fuel-model telemetry table; this keeps the within-model comparison consistent, but it is not a vessel-specific draft measurement for the MarineTraffic AIS vessel.

Figure 3 summarises how the stitched graph edge is evaluated: historical edge geometry and speed define the candidate movement, and the assigned metocean inputs are then used for fuel prediction and, when enabled, threshold-based weather screening. It is a conceptual edge-evaluation schematic, not a geographic map of the Alexandria–Istanbul disconnection case.

### 3.3. Random Forest for Fuel Estimation

To translate physical parameters into an accurate economic cost, an ensemble Machine Learning model, specifically a Random Forest Regressor F(X), is deployed. Random Forests operate by constructing a multitude of decision trees during training. Each tree splits the data based on features to minimise variance. The final output is the mean prediction of the individual trees:

(3) $$F\left(X\right)=\frac{1}{B}\sum_{b=1}^{B}T_{b}\left(X\right),$$

where *B* is the number of trees and $T_{b}\left(X\right)$ is the prediction of the *b*-th tree based on the feature vector *X*. The feature vector for our model is comprised of dynamically changing parameters:

(4) X=[SOG,Mean_Draft,Wave_Height,Wind_Speed].

The Random Forest predicts an hourly fuel rate, which is converted to an edge fuel volume before the graph search. For edge (i,j),

(5) $${\hat{q}}_{ij}=F\left(X_{ij}\right),\;t_{ij}=\frac{d_{ij}}{SOG_{ij}},\;F_{ij}={\hat{q}}_{ij}\;t_{ij},$$

where ${\hat{q}}_{ij}$ is the predicted fuel rate in L/h, $d_{ij}$ is the edge distance in nautical miles, $SOG_{ij}$ is the edge speed in knots, $t_{ij}$ is the traversal time in hours, and $F_{ij}$ is the edge fuel cost in litres.

The target variable in the fuel dataset is already volumetric main-engine consumption, reported as Main engine consumption (L/hr), so no mass-to-volume conversion is applied. For example, if the model predicts ${\hat{q}}_{ij}=800$ L/h for a 10 NM segment travelled at 12.5 kn, then $t_{ij}=10/12.5=0.8$ h and $F_{ij}=800\times 0.8=640$ L.

The model was implemented as a RandomForestRegressor with 100 trees, bootstrap sampling, the squared-error criterion, unrestricted tree depth, all four features considered at each split, minimum split, and leaf sizes of two and one, respectively.

#### Fuel-Model Data and Validation Protocol

The source data comprise 81,342 measured records collected at 60 s intervals from one 81,600-DWT Kamsarmax bulk carrier between January 2021 and February 2023 [7,22]. The journal article [7] is cited for the fuel-consumption modelling context, whereas the Zenodo dataset record [22] is cited for the released data used in the present validation and route-cost experiments.

We used two levels of validation. The first test examines a random 80/20 split: the model is trained on most records from the Zhang et al. vessel and tested on randomly held-out records from the same data source. The stricter tests hide larger row blocks or complete draft regimes before prediction. These tests ask whether the model still works when the test cases are less similar to the training cases; they are not chronological, voyage-wise, or cross-vessel validation due to lack of vessel identifiers.

### 3.4. Dijkstra’s Algorithm and Priority Queues

With the spatial network established and the ML model evaluating costs, the core optimisation relies on Dijkstra’s algorithm. Unlike standard vehicular GPS applications where the weight is geographic distance, the objective here is radically different. The edge weight $W_{i,j}$ is defined as the predicted edge fuel volume in litres, i.e., $W_{i,j}=F_{ij}$ from the rate-to-time conversion above. A route can therefore have a lower predicted fuel cost because of the combined effect of selected geometry, assigned edge speed, draft, and environmental inputs; the contribution of weather alone is not inferred from route-level totals.

The algorithm utilises a Priority Queue (Min-Heap data structure) to efficiently traverse the graph in O(|E|log|V|) time complexity. During the relaxation phase, for every neighboring node *v* of the current node *u*, the algorithm evaluates the following:

(6) $$\mathrm{If}\;Cost\left(u\right)+W_{u,v}<Cost\left(v\right)\Rightarrow Cost\left(v\right)=Cost\left(u\right)+W_{u,v},$$

This finds a path $P^{*}$ that minimises the total predicted fuel consumption across all traversed edges (u,v):

(7) $$P^{*}=\arg\min_{P}\sum_{(u,v)\in P}F_{u,v}$$

where $F_{u,v}$ is the predicted fuel estimate in litres for an edge. This optimality statement is restricted to the constructed static graph, its nonnegative edge weights, the nearest start/end graph nodes, and any active weather-screening rules. It is not a claim of global optimality in continuous maritime space or under unmodelled operational constraints. In the baseline search, wave height and wind speed enter the Random Forest used to estimate $F_{u,v}$ and are not separate penalties. In the experimental weather-screened search, Dijkstra’s algorithm retains the same objective but operates on the subgraph obtained after edges exceeding the selected wave or wind limit have been removed.

## 4. System Implementation and Scalability

This section describes how the methodology in Section 3 is implemented as a modular processing pipeline and how the implementation addresses the computational demands of large AIS graphs, repeated model inference, and external metocean-data requests.

### 4.1. System Architecture

The proposed approach is implemented as a modular, multi-tier pipeline designed to separate data management, environmental-data acquisition, fuel estimation, and route optimisation.

1. Data Ingestion and Storage Layer: A PostgreSQL database, extended with the PostGIS spatial module, acts as the core repository. It stores historical AIS segments kindly supplied by MarineTraffic [23], as well as the World Port Index (WPI) data for origin and destination coordinate mapping [24].
2. Environmental Data Layer: A Python-based module requests current significant wave height and wind speed from the Open-Meteo APIs [25]. These values are used as inputs to fuel prediction.
3. Cognitive Layer (Machine Learning): An offline-trained Random Forest Regressor evaluates the energy requirements of any given vessel state, mapping kinematic and weather variables directly to hourly fuel consumption. The training data are drawn from the published ship-fuel-consumption dataset of Zhang et al. [22].
4. Optimisation Layer: Using the NetworkX library, an undirected graph is traversed using Dijkstra’s algorithm to identify the minimum-predicted-fuel path.

### 4.2. Environmental Data Acquisition and Scenario Control

At execution time, the environmental-data module requests current significant wave height and 10 m wind speed from the Open-Meteo APIs for each required graph location. An edge is represented by its midpoint, whose latitude and longitude are rounded to a one-degree grid before the request is made. This grid is coarse for route screening: one degree of latitude is about 60 NM, while one degree of longitude in the Mediterranean is roughly 42–52 NM depending on latitude. The returned values are stored in the in-memory weather_only_cache, so edges mapped to the same grid cell reuse a single response. In Case Study 1, the 2,116,948 graph edges were represented by 264 weather-grid locations, so each grid value was reused by many graph edges.

For the baseline search, the cached wave and wind values contribute only to the Random Forest fuel estimate. For the sensitivity experiment, the complete grid cache is first populated and then frozen; the same values are reused for every threshold combination while edges exceeding either threshold are removed. This controls the environmental input across scenarios and makes changes in connectivity and route selection attributable to the applied limits. If an API request fails, the implementation assigns fallback values of 1.5 m significant wave height and 8 m/s wind speed to the affected grid cell (the framework queries conditions available at execution time and does not reconstruct conditions corresponding to the timestamps of the historical AIS observations; consequently, the comparison is not a hindcast validation).

### 4.3. Scalability Strategies

Processing millions of AIS data points and executing thousands of ML inferences and API requests introduces substantial computational and network overhead. The implementation addresses these bottlenecks through database-level spatial filtering, indexed nearest-neighbour searches, two-tier caching, and trajectory downsampling. To quantify the implemented components, we also ran a local profiling experiment on 293,939 cargo-polygon records. The experiment is not a comparison with isochrone or time-dependent dynamic-programming solvers; it only measures reproducible components of the proposed implementation. The six-degree corridor proxy retained 211,753 records (72.04%). The BallTree nearest-neighbour search took 0.005 s for 1000 points and 0.045 s for 5000 points, whereas the brute-force nearest-neighbour search took 0.048 s and 1.266 s on the same samples. In synthetic local kNN graphs, NetworkX shortest-path queries were completed in 0.017 s for 1000 nodes/3687 edges and 0.802 s for 25,000 nodes/93,446 edges. These results show that the implemented components are computationally practical at the tested scales. Table 1 summarises the local profiling measurements for the reproducible implementation components. A fair overhead comparison with isochrone or time-dependent dynamic-programming solvers would require implementing those alternatives under the same hardware, software, input data, weather-grid resolution, and route-query conditions, which is left for future work.

We also performed limited component checks rather than a full factorial ablation. Without stitching links, the local Case Study 1 corridor proxy produced 423,255 nodes and 211,638 historical edges split into 211,617 connected components, and no path existed between the nearest start and end graph nodes. With land-screened stitching enabled, the reported graph became connected enough to return the Case Study 1 route analysed in Section 5.

Downsampling reduced the retained AIS reference from 1283 qualifying observations to 152 points (88.2% fewer points), lowering route-reconstruction and plotting cost while preserving the endpoints. Weather-feature influence is examined indirectly through the fuel-model feature-importance diagnostics and through the threshold-screening experiment; caching and spatial indexing are assessed through the profiling results above. A complete ablation of stitching, weather features, caching, and downsampling over many routes is outside the present single-route prototype evaluation and is identified as future work.

#### 4.3.1. Spatial Corridor Queries in PostGIS

Instead of loading the entire network into the system’s memory, the application executes dynamic Spatial Corridor Queries directly within the PostgreSQL backend. Given a route endpoint pair, the system constructs a geometric line string and applies the ST_Buffer command with a generous six-degree limit to form a broad corridor. Subsequently, the ST_Intersects function is utilised to retrieve exclusively those historical AIS segments that intersect this buffer. This dynamic spatial filtering reduces the number of candidate segments passed to graph construction. The six-degree corridor is used as a broad implementation filter around the observed route rather than as a calibrated universal parameter.

#### 4.3.2. BallTree Spatial Indexing

Connecting disconnected historical waypoints to form a cohesive graph using a brute-force approach results in an $O(N^{2})$ time complexity, which is prohibitive for sets with tens of thousands of nodes. To resolve this, the system employs a *BallTree* spatial indexing structure. The BallTree recursively divides the spatial data into nested hyperspheres, allowing the algorithm to execute nearest-neighbour (NN) queries based on the Haversine metric in O(NlogN) time, replacing quadratic brute-force matching with an indexed nearest-neighbour query procedure.

#### 4.3.3. Two-Tier Caching and Downsampling

Fetching weather data and evaluating the fuel model independently for every edge would introduce substantial network and inference latency. The environmental cache described in Section 4.2 forms the first caching tier. A secondary structure (ml_cache) stores Random Forest predictions for rounded tuples of speed, draft, wave height, and wind speed; speed is rounded to the nearest 0.50 knots, draft to 0.001 m, and wave and wind to 0.001 in their respective units. Draft is constant in the present experiments, but it remains part of the model input and the cache key. When another edge requests an existing combination, the cached prediction is retrieved in O(1) time, avoiding both a repeated API request and repeated model inference.

Furthermore, historical AIS trajectories often contain oversampling, loitering noise, and observational gaps. A dynamic downsampling algorithm discards an intermediate point when its Haversine distance from the last retained point is less than 5 NM. This reduces computational demand but does not reconstruct missing observations. As a consequence, long gaps remain straight connections between recorded positions.

## 5. Experimental Results and Discussion

### 5.1. Fuel-Model Validation

Table 2 reports the validation results. In the baseline random split, which reproduces the original 80/20 split, the model predicted similar same-vessel records accurately, with MAE 22.27 L/h, RMSE 38.23 L/h, and $R^{2}=0.966$. The cleaned-data random split and duplicate grouping changed the results only slightly.

To check that the selected Random Forest was not an arbitrary modelling choice, we also compared it with a simple feed-forward neural-network regressor and two lightweight tabular baselines using the same cleaned-data random 80/20 split. Table 3 summarises this model-choice check.

In this tabular single-vessel dataset, the Random Forest achieved the lowest error and highest $R^{2}$ among the tested models, while remaining simple to inspect through feature importance and fast enough for repeated edge-level evaluation. We therefore retained it as the edge-cost model. This result should be read as support for the present implementation, not as a general claim that Random Forests are superior to neural-network fuel models.

The stricter tests were weaker: row-order blocking gave MAE 83.45 L/h and $R^{2}=0.578$, while withholding complete draft regimes gave MAE 154.77 L/h and negative pooled $R^{2}$. Thus, the model is reliable for interpolation within the Zhang et al. vessel data, but these experiments do not prove performance for a different vessel or a completely unseen loading condition.

Feature-importance checks showed that draft and speed were the dominant predictors. Overall, the model predicts randomly held-out records from the same source vessel well, but the stricter holdout tests do not establish transfer to another vessel. Figure 4 provides the error diagnostics used to verify the fuel model, including observed-versus-predicted and residual plots.

To assess the empirical speed domain of the fuel model, we quantified the range represented in the measured fuel-model data. After applying the same model-column completeness filter used for validation, the 81,342 training records covered SOG values from 0.00 to 16.15 kn, with a median of 11.09 kn, a 95th percentile of 13.50 kn, and a 99th percentile of 14.56 kn (Figure 5). These values define the empirical speed domain used for the subsequent route-level out-of-domain diagnostic and capped-speed robustness checks.

Selected routes are examined through two complementary case studies to demonstrate the feasibility and analytical capabilities of the proposed method and to investigate the effects of its principal parameters. The first case study compares a fuel-minimising route with a historical AIS observation sequence for the same endpoints and examines their predicted fuel consumption, travel characteristics, and metocean exposure. This comparison uses common descriptive metrics only. Historical AIS tracks may reflect decisions made under traffic, chart, company, port, and COLREGs constraints [26], but these constraints are not automatically transferred to a newly generated shortest path. The second applies factorial wave- and wind-threshold scenarios to selected routes to assess how the screening parameters affect edge removal, graph connectivity, and route selection. Figure 3 summarises the same edge-evaluation concept used in this second case study: historical edge geometry and speed are combined with the frozen execution-time weather snapshot before edges are retained, removed, or costed. These scenarios also demonstrate how environmental admissibility constraints can support safety-oriented route assessment by excluding edges exposed to conditions above specified limits. However, the experimental limits are not validated vessel-operability thresholds, and satisfying them does not establish the navigational safety of a route. The case studies illustrate the behaviour of the method under controlled conditions rather than provide batch or population-level validation. Fuel values are model predictions, and the environmental inputs represent conditions retrieved at execution time rather than weather-matched hindcasts of the historical voyages.

### 5.2. Case Study 1: Trans-Mediterranean Route Comparison

The first case study used the general cargo ship ORCANA, now CELTIC FORTUNE (MMSI 267242 in the local MarineTraffic AIS extract; the IMO field is recorded as -1 and is therefore unavailable in this dataset). The retained AIS track starts near Ceuta, Spain, at 35.9413 N, 4.9993 W and ends near Ashdod, Israel, at 31.8273 N, 34.6399 E; these are closest-port descriptors rather than verified port-call records. From 1283 qualifying AIS observations, spatial downsampling at 5 NM retained 152 reference-route points. The eco-route was generated between the first and last retained AIS coordinates using a $6^{\circ }$ cargo-graph corridor and a 12 NM stitching threshold. The corridor retains plausible Mediterranean alternatives around the observed route, while the 12 NM stitching threshold connects nearby graph components without allowing long artificial jumps; both values are prototype implementation parameters. The resulting graph contained 425,743 nodes and 2,116,948 edges.

To check whether the 12 NM stitching threshold alone determined the Case Study 1 path, we ran an offline sensitivity diagnostic using the same local SQL-dump corridor proxy, fixed weather values of 1.5 m wave height and 8.0 m/s wind speed, and land-screened stitching candidates up to 15 NM. This diagnostic isolates the graph-stitching parameter and is not a live-weather rerun of the manuscript baseline. Routes were found for all tested thresholds, and the selected route metrics were unchanged from 8 to 15 NM; the largest stitching edge actually used by the selected route was 4.85 NM. Thus, in this corridor proxy, the reported route is not dependent on permitting links close to the 12 NM limit, although the threshold remains a prototype calibration parameter because stitching supplies most of the path connectivity. Table 4 summarises this stitching-threshold diagnostic.

The historical AIS reference route and the generated eco-route were evaluated with the same pretrained Zhang et al. Random Forest and shared execution-time weather cache. The draft input was also common to both route alternatives, whereas speed followed the route representation: recorded AIS speed for retained AIS-reference segments and stored historical graph-edge speed for the generated route. The fuel model was trained on telemetry from an 81,600-DWT Kamsarmax bulk carrier, whereas the AIS route is for a general cargo vessel; the common 11.264 m draft input comes from the fuel-model source data and is not a verified operating draft for ORCANA/CELTIC FORTUNE. The AIS reference route covered 2044.70 NM in an estimated 183.44 h and had a model-estimated fuel total of 160,230 L. The fuel-minimising eco-route covered 2235.10 NM in 162.08 h and had a model-estimated total of 119,312 L. Thus, relative to the AIS reference, the generated route was 9.31% longer, had an 11.65% shorter reconstructed travel time, and had 25.54% lower model-estimated fuel. This baseline percentage is an uncontrolled within-model result under different speed assignments and cross-vessel model transfer: recorded AIS speeds for the reference sequence and graph-inherited historical edge speeds for the generated route. It should therefore be interpreted together with the speed-domain robustness checks below, not as an independent validation, measured saving, or operational guarantee.

Figure 6 visualises the paired trajectories. The eco-route is selected by minimum predicted fuel within the available graph, not by minimum distance or a weather-exposure objective.

#### Comparative Metocean Exposure

Table 5 compares distance-weighted metocean descriptors for the two routes. The eco-route had a higher mean wave height (0.65 m versus 0.57 m) and mean wind speed (5.41 m/s versus 4.85 m/s). Both routes had a maximum wave height of 1.5 m, while the maximum wind speed was 9 m/s on the eco-route and 8 m/s on the AIS reference. Under this execution-time weather snapshot, the lower baseline predicted fuel therefore did not coincide with lower distance-weighted metocean exposure; this should not be interpreted as a general physical relationship. The averages in Table 5 are route-level aggregates over different edge sequences, not a same-edge comparison. An edge-level diagnostic gave AIS-reference speeds centred at 11.2 kn (IQR 11.0–11.4) and generated-route speeds centred at 16.6 kn (IQR 14.7–18.5), with the same draft input of 11.264 m in both cases. The generated route also included many edge speeds above the fuel-model training speed range, so the lower baseline fuel total should be read as a within-model shortest-path result with speed-assignment and extrapolation risk, not as evidence that higher speed alone reduces hourly fuel use.

To test this mechanism, we re-evaluated the selected route geometries using the archived edge-level diagnostic behind the speed distributions. The diagnostic is not a new graph optimisation: it keeps the selected AIS-reference and generated-route geometries fixed and changes only the speed inputs used for Random Forest prediction and distance/speed traversal time. The AIS reference had no segment above the fuel-model training maximum speed of 16.15 kn. In contrast, the generated route had 708 of 1231 segments above that maximum, corresponding to 1749.94 NM or 79.53% of its diagnostic distance; 90.52% of its distance exceeded the training 99th percentile speed of 14.56 kn.

The robustness results show that the central fuel comparison is highly sensitive to the generated-route speed assignment. Capping generated-route speeds to the training maximum reduced the diagnostic fuel advantage from 29.10% to 19.76%, and capping to the training 99th percentile reduced it further to 12.34%. When both selected geometries were evaluated at the AIS-reference aggregate speed of 11.15 kn, the generated route became 7.61% higher in model-estimated fuel because it is longer. Thus, the baseline 25.54% result should be presented as the output of the original speed-assigned graph-cost formulation, not as a paired geometry-only fuel saving. Table 6 reports these fixed-geometry speed-domain robustness checks.

Table 7 reports the distance and fraction of each route above the four experimental threshold pairs. The exposure columns refer to the fixed AIS-reference and eco-route geometries from Table 5, not to newly optimised screened routes. The “Route” column reports whether a graph path remained after applying the corresponding weather-only exclusions; for rows marked Found, the baseline route metrics remain those reported in Table 5. At the Low thresholds, the eco-route reduced wave exposure from 136.18 NM (6.66%) to 56.84 NM (2.54%), but introduced 167.66 NM (7.50%) of wind exposure compared with none on the AIS route. The same eco-route wind exposure remained at Medium thresholds 1 and decreased to 112.57 NM (5.04%) at Medium thresholds 2. Neither route exceeded the High thresholds. Therefore, the exposure response is variable-specific and does not support a general claim that the eco-route avoids adverse weather.

The shared snapshot covered 264 weather-grid locations. These 264 rounded one-degree locations represented the 2,116,948 graph edges used in Case Study 1. Complete wave and wind data were available for 236 locations; the remaining 28 used fallback values of 1.5 m and 8 m/s. Open-Meteo returned 264 marine responses and 263 atmospheric responses. A value was counted as an exceedance only when it was strictly greater than the corresponding threshold; therefore, fallback values equal to a threshold were not counted. These exposure measures describe conditions at execution time, not at the historical AIS timestamps, and should not be interpreted as a navigational-risk or safety assessment.

The AIS observations span 13–31 May 2016 and are not temporally continuous: the maximum gap between qualifying observations was 140.80 h, and the largest straight segment in the downsampled sequence was 487.39 NM. Consequently, the reported AIS travel time is reconstructed as the sum of segment distance divided by recorded speed rather than measured elapsed voyage time. Weather assigned to long missing-data segments is likewise a coarse spatial approximation. For both compared routes, travel time is estimated from edge distance and historical edge speed, while fuel consumption is estimated from distance, speed, and the pretrained fuel model under the same execution-time weather snapshot. The paired results should therefore be interpreted as a prototype-level exposure comparison, not as validation against a complete operational voyage record.

### 5.3. Case Study 2: Sensitivity to Wave and Wind Thresholds

The second case study used a controlled factorial experiment to test how weather screening affects graph connectivity and the fuel-minimising path. For each route, one cargo-vessel graph was constructed with a $6^{\circ }$ corridor and a 12 NM stitching threshold, and all nine combinations of wave limits {1.2,1.5,2.0} m and wind limits {8,8.5,12} m/s were evaluated using one frozen execution-time weather snapshot. Edges were excluded sequentially by wave and then wind. The limits are experimental sensitivity parameters, not validated vessel-operability or navigational-safety limits. Table 8 reports the fuel-only baseline path for each controlled batch before applying weather screening. These baselines provide the reference path length, reconstructed time, predicted fuel, and metocean exposure against which the threshold experiments are interpreted. The Path edges column counts only edges along the selected baseline route; graph-level edge removals are reported separately in Table 9.

In Table 9, N denotes that no feasible route remained after screening, and U denotes that the feasible screened route was unchanged from the fuel-only baseline. The exclusion counts in this table are graph-level removals caused by the wave and wind filters, so they are not intended to match the path-edge counts in Table 8. Table 9 then summarises the full factorial screening results, showing how many graph edges were removed by each wave–wind threshold pair and whether a feasible path still existed.

On Alexandria–Istanbul, every 1.2 m wave-limit combination disconnected the graph, including the 12 m/s wind case; wave screening alone was therefore sufficient for this outcome. At wave limits of 1.5 and 2.0 m, all screened routes were feasible but identical to the baseline: 585 shared edges, complete baseline-edge overlap, and an edge-set Jaccard similarity of 1.0. Their distance, reconstructed time, and predicted fuel consequently remained 833.96 NM, 81.69 h, and 46,314 L. On Piraeus–Limin Sirou, all nine combinations retained the same 91.07 NM, 10.30 h, and 4642 L baseline path with the same similarity values, despite removing up to 138,356 edges. Thus, this snapshot produced two outcomes—disconnection or an unchanged optimum—and no feasible alternative route from which to estimate a fuel, distance, or time trade-off.

The coarse one-degree weather grid is a likely contributor to this binary behaviour. Because many graph edges share the same rounded weather cell, threshold screening removes or retains spatial blocks of edges rather than producing fine-grained edge-level variation. If a low threshold removes grid cells that act as graph bridges, the route can disappear entirely; if all grid cells along the baseline optimum remain below the active threshold, the fuel-minimising path can remain unchanged even after many off-path edges are removed. Table 9 should therefore be interpreted as a single-snapshot, grid-resolution-dependent sensitivity check, not as evidence that weather screening generally produces only disconnected or unchanged routes. The archived Table 9 records preserve aggregate exclusion counts and route outcomes, but not persistent edge-level component geometries sufficient to reconstruct a static Alexandria–Istanbul disconnection map for the 1.2 m wave-limit cases; regenerating such a map with live API calls would create a different weather snapshot.

Both batches passed the validation requirement that every feasible screened route had zero distance above its active limits. The Alexandria–Istanbul batch requested 142 weather-grid locations, of which 116 were complete Open-Meteo values and 26 used the fixed fallback; its baseline traversed 45 fallback-valued edges covering 45.29 NM. When fallback-affected edges were excluded rather than assigned 1.5 m and 8 m/s, all nine Alexandria–Istanbul cases became disconnected. The Piraeus–Limin Sirou batch requested 115 locations, with 100 complete and 15 fallback locations; its baseline used no fallback-valued edge and remained unchanged under fallback-edge exclusion. The primary-route conclusion is therefore dependent on missing-weather treatment, whereas the shorter-route conclusion is not. These single-snapshot results characterise graph sensitivity only and do not validate navigational safety or temporal robustness.

### 5.4. Limitations, Operational Role, and Safety Extensions

Although the case studies demonstrate the potential of the proposed approach, their findings must be interpreted within the scope of the current prototype. The system is intended for research decision support and does not approve or certify navigational routes. Historical AIS segments indicate prior use, while the sampled land-mask test provides only a coarse check of newly created links. Neither establishes operational safety. The prototype does not currently represent bathymetry, under-keel clearance, charted hazards, restricted areas, traffic-separation schemes, navigational notices, currents, vessel responses, traffic encounters, collision risk, or COLREGs compliance. Final route planning therefore remains the responsibility of qualified personnel using authoritative charts, regulations, current traffic information, and vessel-specific guidance. Thus, the AIS comparison is reasonable as a prototype-level fuel, distance, reconstructed-time, and exposure comparison, but not as proof that the generated route is operationally navigable or COLREGs-compliant. If COLREGs-related or navigational constraints are available as spatial layers or edge attributes, such as restricted areas or traffic-separation schemes, the same graph-filtering mechanism used here for land-crossing checks can remove non-compliant edges before the shortest-path search. A remaining limitation is that dynamic vessel-encounter COLREGs compliance requires time-aligned traffic data and an explicit encounter model. Practical use would therefore require an additional validation layer that checks candidate routes against electronic nautical charts, traffic-separation schemes, restricted areas, bathymetry, forecast metocean fields, real-time AIS traffic, vessel-specific operating limits, and COLREGs-based encounter assessment before presentation to bridge teams or shore-side operators.

Fuel-model validation is also limited by transferability. The measured fuel data used for validation come from the Zhang et al. dataset, while the AIS data used in the route comparison do not include measured fuel consumption or the vessel-specific operating details needed for direct same-vessel validation. More specifically, the source fuel data describe an 81,600-DWT Kamsarmax bulk carrier, whereas Case Study 1 applies the resulting model to a general cargo vessel AIS route. The 11.264 m draft input used for both route alternatives is inherited from the fuel-model source data and should not be interpreted as a verified ORCANA/CELTIC FORTUNE draft. The reported route totals therefore apply the same pretrained model to both route alternatives and should be read as a within-model comparison. They do not verify absolute fuel use, cross-vessel accuracy, cross-ship-type accuracy, or a realized 25.54% saving. Vessel-specific fuel telemetry would be required for such a claim.

The paired fuel result is also limited by the observed speed domain. Random Forest predictions are interpolative over the training samples and do not provide physically reliable extrapolation beyond the measured SOG range. The selected generated-route diagnostic therefore remains a within-model comparison rather than an independent validation of absolute performance. A fuller remedy would rerun the graph search with out-of-domain speeds capped, penalised, or excluded before Dijkstra; the present revision re-evaluates the already selected geometries and reports this as a robustness diagnostic.

The metocean analysis is also limited by the use of execution-time Open-Meteo snapshots and a one-degree weather grid rather than time-matched reanalysis. A historical ERA5-style hindcast would require matching each AIS and generated-route segment to a traversal time and interpolating historical fields in both space and time. Because the current AIS reference has large temporal gaps and the generated route is a reconstructed graph path rather than an observed voyage, the present results are restricted to snapshot-based route assessment. The one-degree grid can also amplify threshold effects by assigning identical wave and wind values to many nearby graph edges, which helps explain why the weather-screening experiment yielded only disconnection or an unchanged optimum under this snapshot.

Reproducibility is also limited by the AIS fields available for Case Study 1. The local extract does not itself contain verified vessel name, destination port, deadweight, length, draft range, engine rating, or measured fuel consumption. The case study is therefore reproducible at the level of the AIS-derived route, model inputs, graph parameters, and algorithm configuration, but not as a vessel-specific engineering trial with independently measured fuel use. For this revision, we also performed a streaming continuity scan of the local AIS extract. The scan found other Mediterranean cargo tracks with smaller observation gaps under a rule of at least 100 observations, maximum gap at most 24 h, maximum segment at most 100 NM, and endpoint distance at least 500 NM. These candidates have different vessels and endpoints, however, so adding them would require a separate graph-construction, weather-assignment, fuel-evaluation, and screening batch rather than a direct replacement of Case Study 1; this broader multi-case validation is left for future work.

Computational benchmarking is another limitation. The local component-profile results above support the practicality of the implemented corridor filtering, BallTree stitching, cached weather/fuel evaluations, and shortest-path search, but they do not establish a measured computational advantage over traditional weather-routing algorithms. The same edge-filtering structure can incorporate static navigational restrictions when they are available as spatial layers or edge attributes, but dynamic vessel-encounter COLREGs compliance still requires time-aligned AIS traffic and an explicit encounter model. Specific runtime and memory comparisons against reimplemented isochrone, dynamic-programming, and alternative graph-based solvers are therefore treated as future work.

The existing architecture nevertheless provides a direct path for incorporating authoritative safety information. The PostGIS-backed graph can associate candidate edges with spatial attributes and exclude or flag unsuitable edges before Dijkstra’s fuel-minimisation search. A first extension would sample depth from a versioned bathymetric layer and compare it with vessel draft, predicted water level, and a documented under-keel-clearance margin. EMODnet bathymetry can support reproducible research-scale development in European waters [27]; operational use, however, requires current ENCs issued by authorised hydrographic offices [28].

Further edge attributes could represent charted hazards, restricted areas, traffic-separation schemes, and navigational notices. Dynamic traffic assessment would require time-aligned AIS data, closest-point-of-approach calculations, and an explicit COLREGs decision model [19,26]. Documented vessel-specific wave and wind operability limits could also be introduced as edge constraints. The resulting safety extensions must be validated against expert-reviewed or operationally approved routes before supporting any operational-safety claim.

## 6. Conclusions and Future Work

This paper presented an AIS-informed, land-aware eco-routing prototype for maritime decision support. Historical AIS segments constrain the graph, newly created stitching links are screened against a land mask, and a Random Forest model estimates edge fuel cost from vessel and current metocean inputs. In the illustrative paired experiment, the eco-route had lower model-estimated fuel and reconstructed travel time but was longer and had higher mean wave and wind exposure than the AIS reference sequence. The paired comparison should therefore be read as a within-model diagnostic: the lower baseline fuel value was associated with higher assigned generated-route speeds, and when both selected geometries were re-evaluated at the AIS-reference speed, the generated route had higher model-estimated fuel because of its longer distance. Fuel-model tests showed strong random-split interpolation but substantially weaker row-block performance and no demonstrated extrapolation to an unseen draft regime. The controlled factorial study further showed that experimental weather limits can remove many edges without changing the fuel-optimal path, or can disconnect the available graph without yielding an alternative route; the primary-route outcome was also sensitive to fallback-weather treatment. These findings emphasise that fuel minimisation, speed assignment, metocean exposure, graph connectivity, and missing-weather assumptions are distinct considerations. Because this study uses one incomplete AIS sequence without measured vessel fuel or reliable vessel particulars, a model trained on one source vessel, and execution-time weather snapshots, it does not establish measured or general fuel savings, cross-vessel accuracy, temporal robustness, or operational safety performance.

Building on the edge-level safety-coverage roadmap above, future work will add time-dependent forecasts, vessel-response models, COLREGs-aware encounter checks, and multi-objective optimisation so that operators can examine reproducible trade-offs between fuel, arrival time, weather exposure, and explicitly defined safety constraints. Future route-cost experiments should also enforce the fuel-model training domain during graph construction, for example by capping, penalising, or excluding out-of-domain speeds before optimisation. Future work will also include controlled computational-complexity experiments against traditional voyage-optimisation baselines using common hardware, weather data, graph or grid resolutions, and route-query sets.

## Figures and Tables

**Figure 1:**
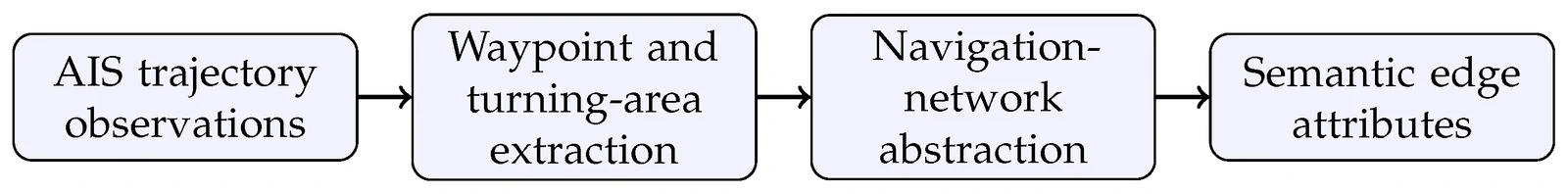
Schematic summary of the AIS-derived navigation-network construction concept used to constrain graph-based route generation.

**Figure 2:**
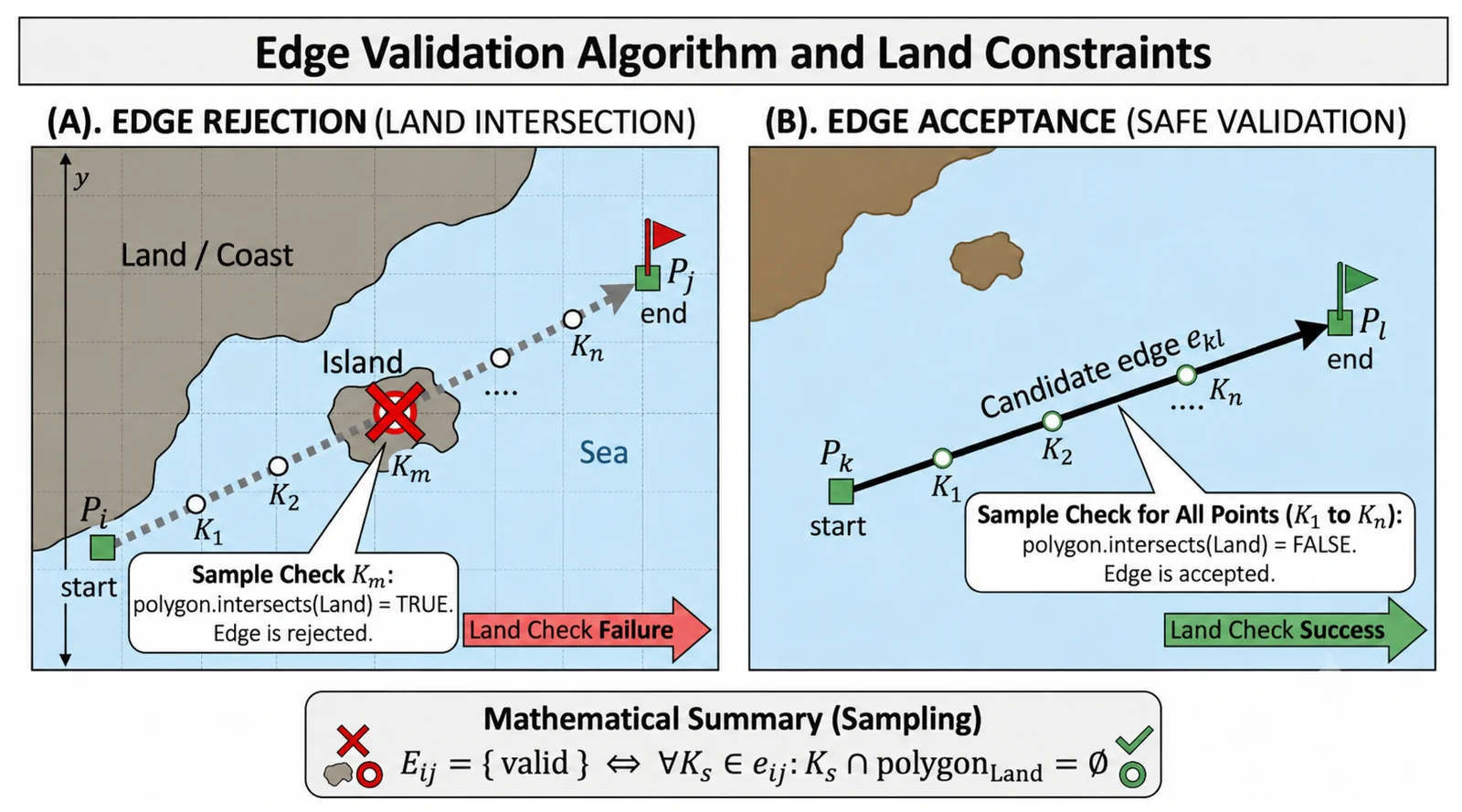
Illustration of the land-mask screen used for newly created graph-stitching links. The figure visualises the implemented overlap check: candidate links whose retained sample points are classified as water are accepted, whereas links with sampled interior points classified as land are rejected.

**Figure 3:**
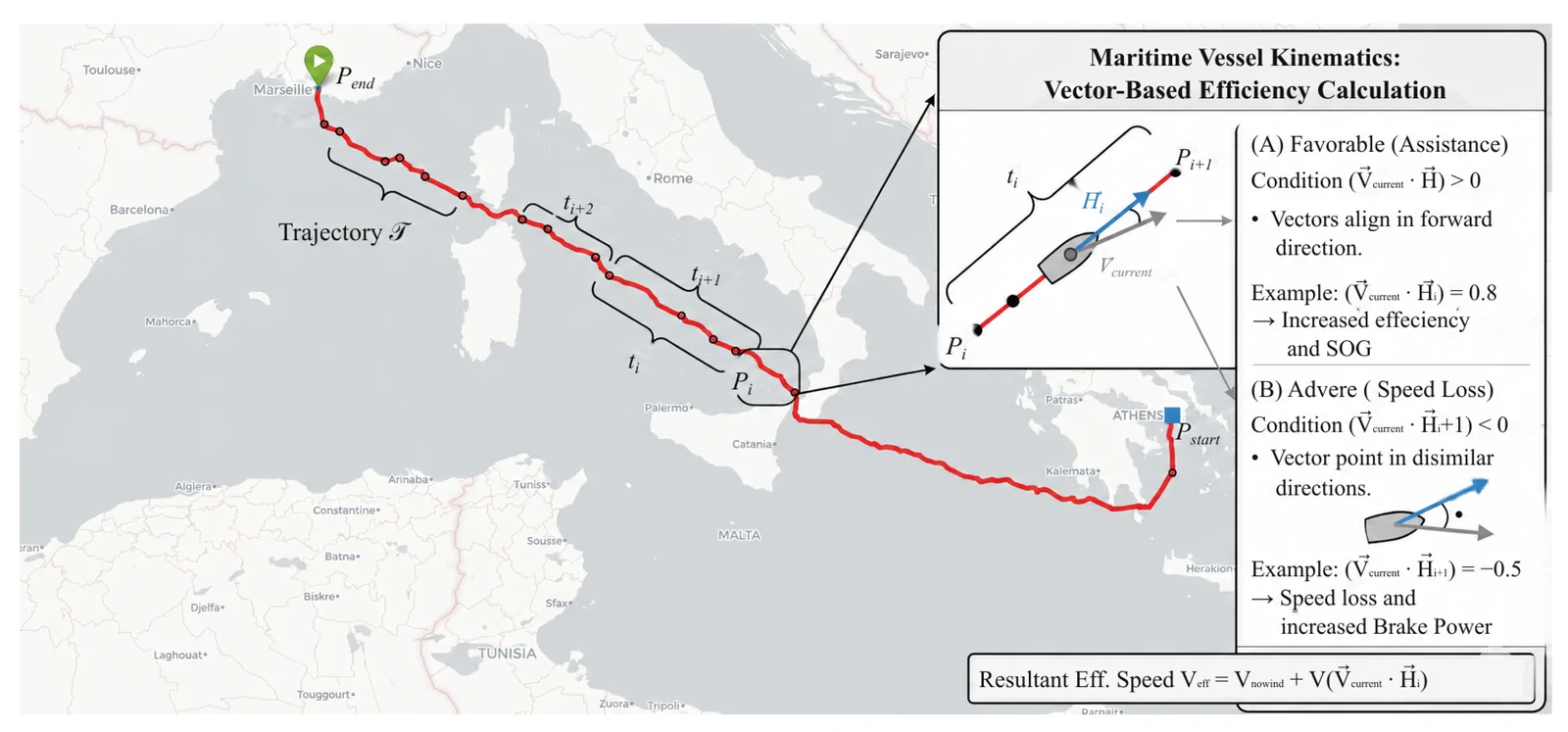
Conceptual illustration of graph stitching and edge evaluation. Historical edge information provides speed, direction, and distance, while execution-time wave and wind conditions are assigned to the edge for fuel prediction and for the Case Study 2 weather-screening tests.

**Figure 4:**
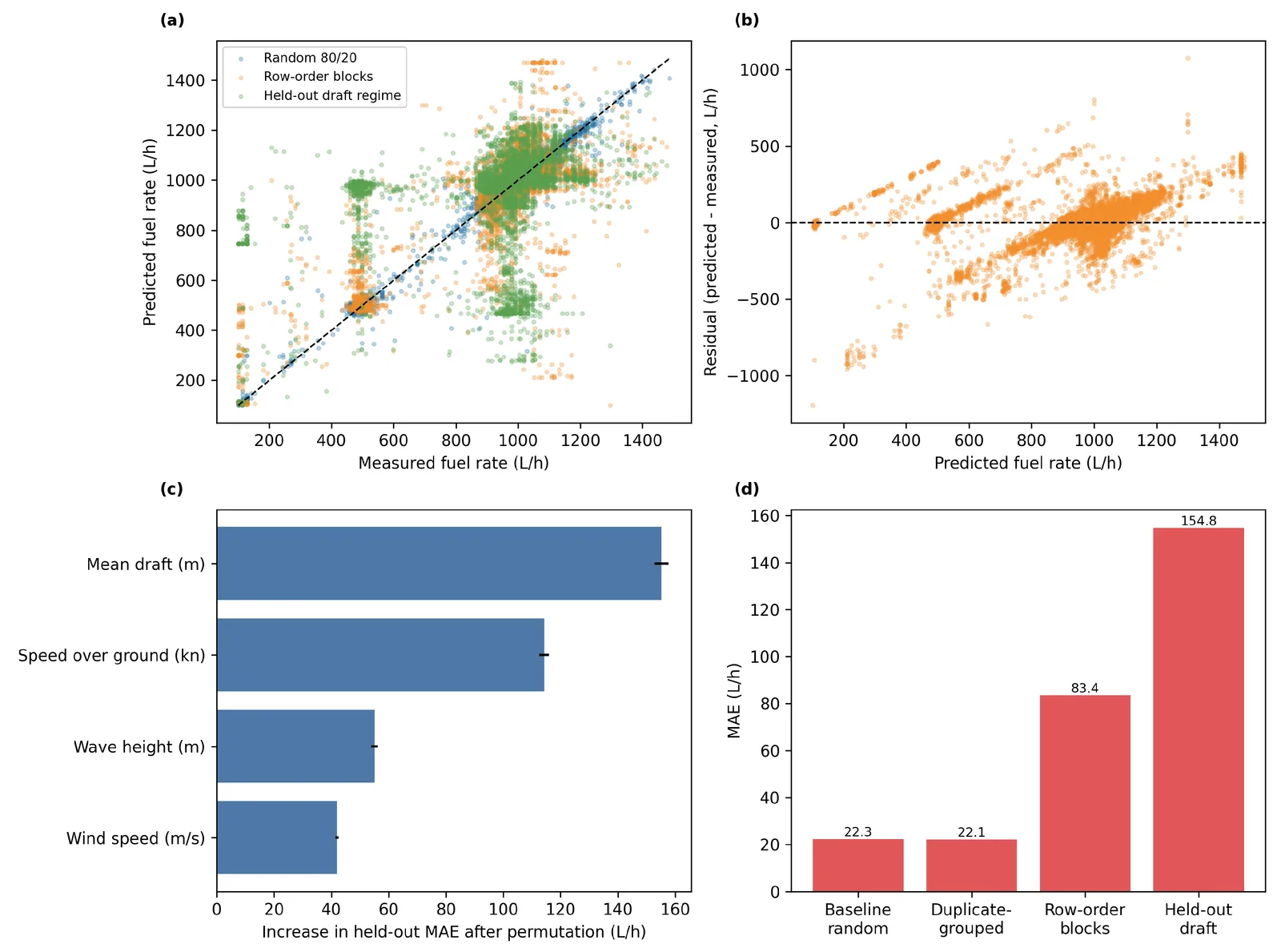
Fuel-model validation diagnostics: (**a**) measured versus predicted fuel rate; (**b**) residuals for the stricter row-order test; (**c**) held-out permutation importance measured as increase in MAE; and (**d**) Random Forest MAE by validation design. The stricter tests show the limit of the model’s transfer interpretation.

**Figure 5:**
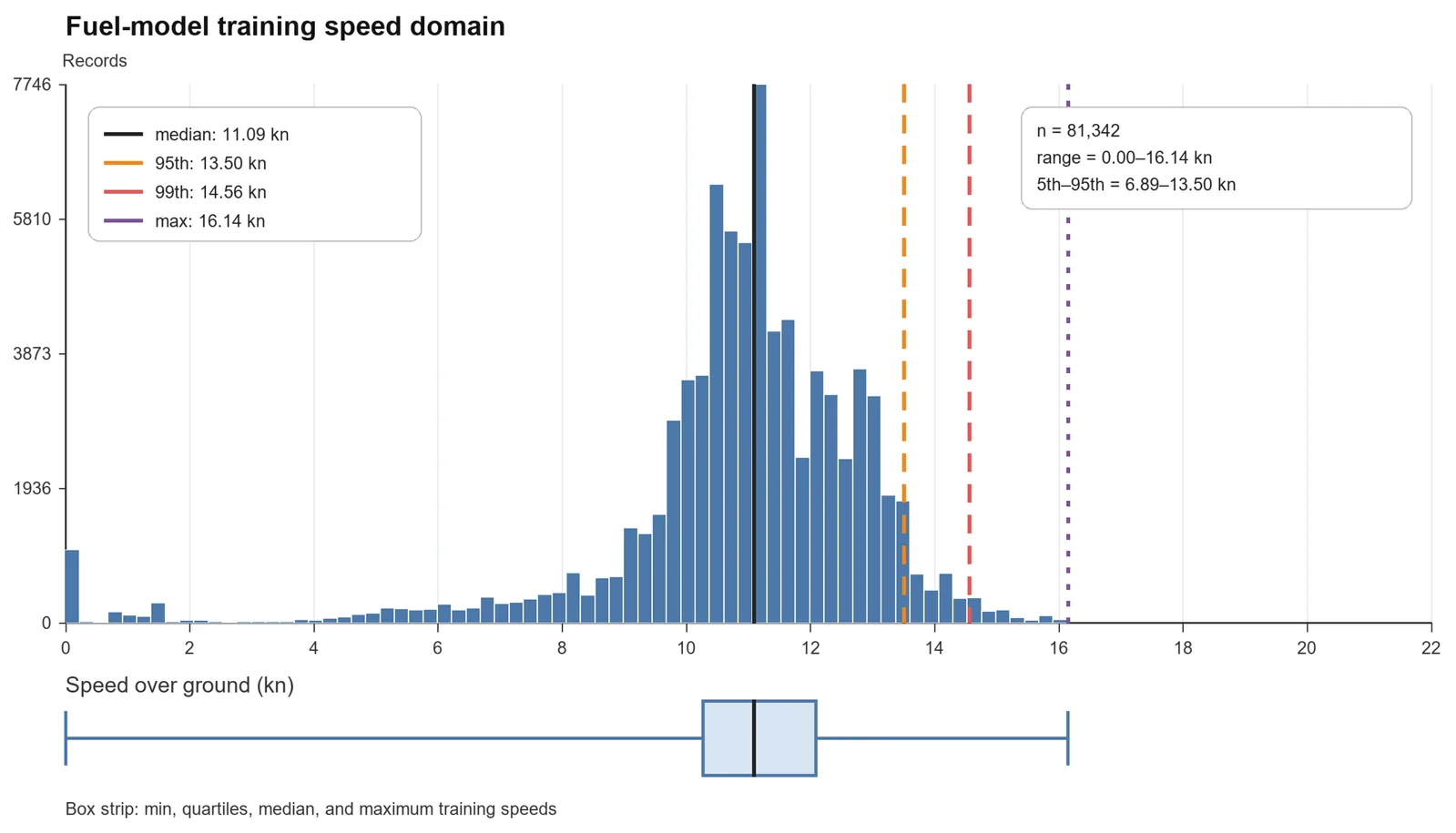
Training speed domain of the measured fuel-consumption dataset used by the Random Forest. The histogram and box strip show that most records are concentrated around 10–13 kn, while the maximum observed SOG is 16.15 kn.

**Figure 6:**
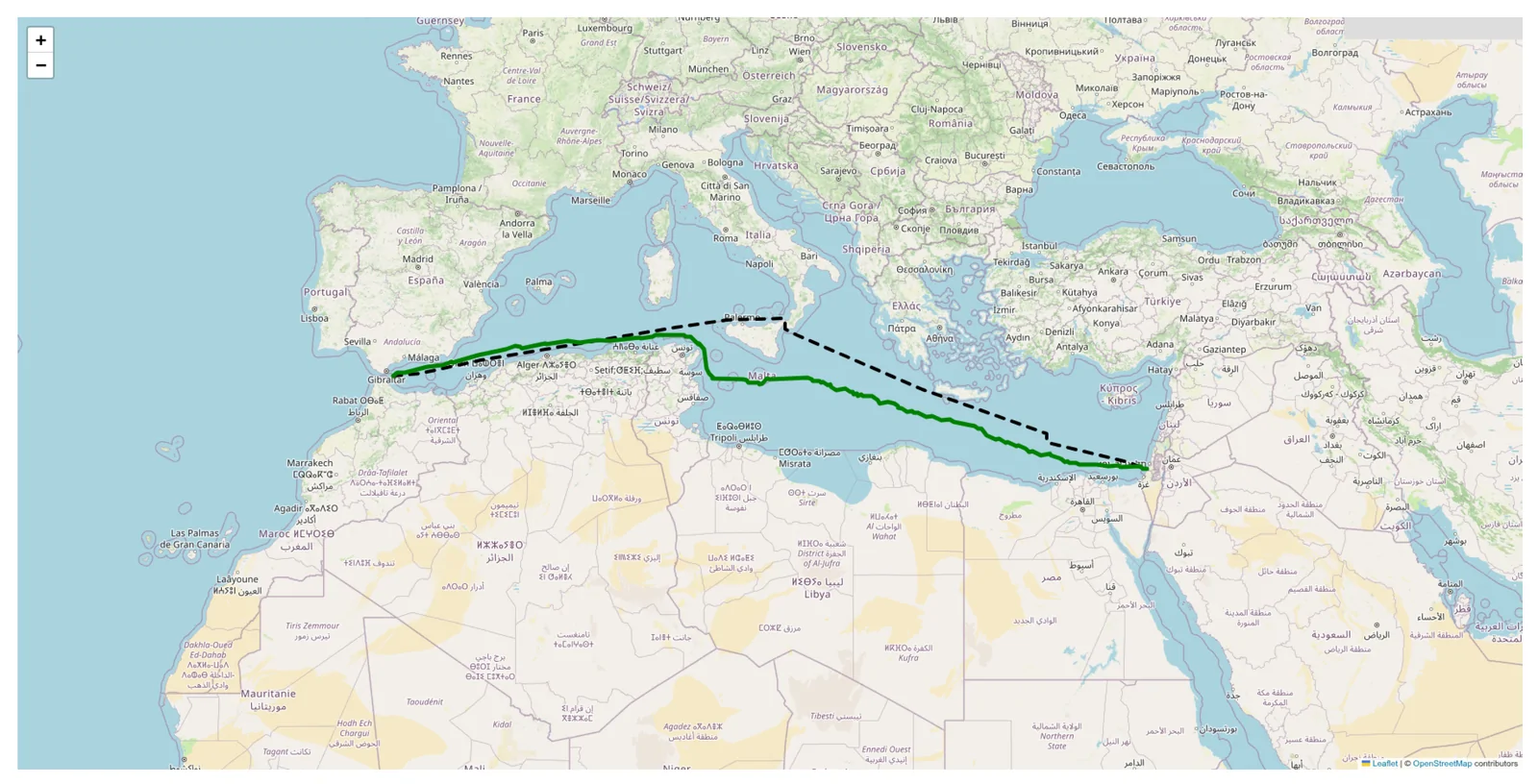
Visual comparison between the downsampled historical AIS reference sequence (black dashed line) and the fuel-minimising eco-route (green line). Long straight AIS segments reflect gaps between observations.

**Table 1:** Local component-level profiling of reproducible implementation steps.

Component	Size	Time (s)	Note
Read cargo-polygon file	293,939 records	0.69	About 65.0 MB in memory
Parse polygon centroids	293,939 records	6.72	Local parsing overhead
six-degree corridor proxy	293,939 records	0.02	211,753 records retained (72.04%)
BallTree nearest-neighbour search	1000/5000 points	0.005/0.045	Brute force took 0.048/1.266 s
BallTree nearest-neighbour search	10,000/25,000/50,000 points	0.126/0.407/1.013	Brute force not run at these sizes
NetworkX shortest path on kNN graph	1000/25,000 nodes	0.017/0.802	3687/93,446 edges

**Table 2:** Random Forest fuel-rate validation. The first rows are simple same-source holdout tests. The row-order and held-out-draft rows are stricter sensitivity tests because the released CSV has no timestamps, voyage IDs, or vessel IDs.

Validation Design	Test *n*	MAE (L/h)	RMSE (L/h)	$R^{2}$	Bias (L/h)	NMAE (%)
Baseline random 80/20	16,114	22.27	38.23	0.966	−0.04	2.36
Cleaned-data random 80/20	16,269	22.51	39.89	0.964	0.34	2.38
Duplicate-grouped 80/20	16,363	22.10	39.86	0.967	0.50	2.35
Five row-order blocks	81,342	83.45	138.44	0.578	4.67	8.85
Leave one draft regime out	81,342	154.77	236.58	−0.231	2.17	16.41

**Table 3:** Fuel-rate model comparison on the same cleaned-data random 80/20 split. The comparison supports the model choice for this prototype and is not intended as a complete benchmark of fuel-consumption models.

Model	MAE (L/h)	RMSE (L/h)	$R^{2}$	Bias (L/h)	Fit Time (s)
Random Forest	22.51	39.89	0.9639	0.34	2.87
Histogram Gradient Boosting	26.55	43.71	0.9566	0.36	2.23
DNN/MLP	55.63	82.59	0.8452	−0.35	14.68
Linear Regression	97.35	128.87	0.6230	0.97	0.03

**Table 4:** Stitching-threshold sensitivity for Case Study 1 using the local fixed-weather corridor diagnostic. Fuel values are model predictions under fixed weather values and are used only to compare the stitching parameter.

Threshold (NM)	Historical Edges	Stitch Edges	Route Found	Path Edges	Distance (NM)	Time (h)	Fuel (L)
8	211,638	1,891,128	Yes	1231	2200.26	124.12	126,464
10	211,638	1,892,383	Yes	1231	2200.26	124.12	126,464
12	211,638	1,892,832	Yes	1231	2200.26	124.12	126,464
15	211,638	1,893,082	Yes	1231	2200.26	124.12	126,464

**Table 5:** Paired route performance and distance-weighted metocean descriptors. Fuel values are model predictions.

Route	Distance (NM)	Time (h)	Fuel (L)	Mean Wave (m)	Max Wave (m)	Mean Wind (m/s)	Max Wind (m/s)
AIS reference	2044.70	183.44	160,230	0.57	1.5	4.85	8
Eco-route	2235.10	162.08	119,312	0.65	1.5	5.41	9

**Table 6:** Speed-domain robustness of the paired route comparison using the archived edge-level diagnostic. Fuel values are model re-evaluations of fixed selected route geometries; this table does not rerun Dijkstra after capping or excluding high-speed graph edges.

Scenario	Route	Distance (NM)	Time (h)	Fuel (L)	Mean Speed (kn)	Max Speed (kn)	Generated Fuel Difference
Original archived edge assignment	AIS reference	2044.70	183.44	178,448	11.17	12.90	–
Original archived edge assignment	Generated route	2200.26	124.12	126,518	18.13	23.04	−29.10%
Common AIS-reference speed	AIS reference	2044.70	183.44	178,744	11.15	11.15	–
Common AIS-reference speed	Generated route	2200.26	197.40	192,342	11.15	11.15	+7.61%
Generated speed capped at training max	AIS reference	2044.70	183.44	178,448	11.17	12.90	–
Generated speed capped at training max	Generated route	2200.26	140.39	143,181	15.77	16.14	−19.76%
Generated speed capped at training 99th percentile	AIS reference	2044.70	183.44	178,448	11.17	12.90	–
Generated speed capped at training 99th percentile	Generated route	2200.26	153.15	156,435	14.40	14.56	−12.34%

**Table 7:** Route exposure and weather-only graph screening. Exposure entries are NM (percentage of the fixed Table 5 route distance); edge exclusions are graph-level wave/wind counts applied sequentially, and Found means that a path remained after screening.

Scenario/ Thresholds	Wave (m)	Wind (m/s)	AIS Wave	AIS Wind	Eco Wave	Eco Wind	Excluded Edges	Route
Low	1.2	8	136.18 (6.66%)	0.00 (0.00%)	56.84 (2.54%)	167.66 (7.50%)	106,615/66,769	Not found
Medium 1	1.5	8	0.00 (0.00%)	0.00 (0.00%)	0.00 (0.00%)	167.66 (7.50%)	0/66,769	Found
Medium 2	1.5	8.5	0.00 (0.00%)	0.00 (0.00%)	0.00 (0.00%)	112.57 (5.04%)	0/47,508	Found
High	2.0	12	0.00 (0.00%)	0.00 (0.00%)	0.00 (0.00%)	0.00 (0.00%)	0/0	Found

**Table 8:** Fuel-only baselines for the two controlled weather-screening batches. Path edges are the edges in the selected baseline path, not the number of graph edges available before screening. Fuel values are model predictions.

Route	Path Edges	Distance (NM)	Time (h)	Fuel (L)	Mean/Max Wave (m)	Mean/Max Wind (m/s)	Fallback Edges	Fallback Distance (NM)
Alexandria–Istanbul	585	833.96	81.69	46,314	0.43/1.5	4.05/8	45	45.29
Piraeus–Limin Sirou	99	91.07	10.30	4642	0.17/0.38	2.58/2.69	0	0.00

**Table 9:** Full factorial weather-screening results. Each cell reports sequential wave/wind edge exclusions followed by route status: N denotes no route and U denotes a feasible route unchanged from the fuel-only baseline.

Route	Wave Limit (m)	Wind 8 m/s	Wind 8.5 m/s	Wind 12 m/s
Alexandria–Istanbul	1.2	67,783/56,976; N	67,783/46,629; N	67,783/0; N
Alexandria–Istanbul	1.5	0/65,801; U	0/55,454; U	0/0; U
Alexandria–Istanbul	2.0	0/65,801; U	0/55,454; U	0/0; U
Piraeus–Limin Sirou	1.2	66,998/71,358; U	66,998/47,301; U	66,998/0; U
Piraeus–Limin Sirou	1.5	0/88,448; U	0/64,391; U	0/0; U
Piraeus–Limin Sirou	2.0	0/88,448; U	0/64,391; U	0/0; U

## Data Availability

The data and code supporting the findings of this study are available from the corresponding author upon reasonable request.

## References

[1] UN Trade and Development (2025). Review of Maritime Transport 2025: Staying the Course in Turbulent Waters. https://unctad.org/publication/review-maritime-transport-2025.

[2] International Maritime Organization (2026). AIS Transponders. https://www.imo.org/en/ourwork/safety/pages/ais.aspx.

[3] Harati-Mokhtari A., Wall A., Brooks P., Wang J. (2007). Automatic Identification System (AIS): Data Reliability and Human Error Implications. J. Navig..

[4] Pallotta G., Vespe M., Bryan K. (2013). Vessel Pattern Knowledge Discovery from AIS Data: A Framework for Anomaly Detection and Route Prediction. Entropy.

[5] Tu E., Zhang G., Rachmawati L., Rajabally E., Huang G.B. (2018). Exploiting AIS Data for Intelligent Maritime Navigation: A Comprehensive Survey from Data to Methodology. IEEE Trans. Intell. Transp. Syst..

[6] Varlamis I., Kontopoulos I., Tserpes K., Etemad M., Soares A., Matwin S. (2021). Building navigation networks from multi-vessel trajectory data. GeoInformatica.

[7] Zhang M., Tsoulakos N., Kujala P., Hirdaris S. (2024). A Deep Learning Method for the Prediction of Ship Fuel Consumption in Real Operational Conditions. Eng. Appl. Artif. Intell..

[8] Zis T.P., Psaraftis H.N., Ding L. (2020). Ship weather routing: A taxonomy and survey. Ocean Eng..

[9] Walther L., Rizvanolli A., Wendebourg M., Jahn C. (2016). Modeling and optimization algorithms in ship weather routing. Int. J. E-Navig. Marit. Econ..

[10] Zaccone R., Ottaviani E., Figari M., Altosole M. (2018). Ship voyage optimization for safe and energy-efficient navigation: A dynamic programming approach. Ocean Eng..

[11] Chen Y., Zhang C., Guo Y., Wang Y., Lang X., Zhang M., Mao W. (2025). State-of-the-art optimization algorithms in weather routing - ship decision support systems: Challenge, taxonomy, and review. Ocean Eng..

[12] Lu R., Turan O., Boulougouris E., Banks C., Incecik A. (2015). A semi-empirical ship operational performance prediction model for voyage optimization towards energy efficient shipping. Ocean Eng..

[13] Kaklis D., Eirinakis P., Giannakopoulos G., Spyropoulos C., Varelas T.J., Varlamis I. (2022). A big data approach for Fuel Oil Consumption estimation in the maritime industry. Proceedings of the 2022 IEEE Eighth International Conference on Big Data Computing Service and Applications (BigDataService).

[14] Vettor R., Soares C.G. (2016). Development of a ship weather routing system. Ocean Eng..

[15] Mannarini G., Coppini G., Oddo P., Pinardi N. (2013). A prototype of ship routing decision support system for an operational oceanographic service. TransNav Int. J. Mar. Navig. Saf. Sea Transp..

[16] Wei S., Zhou P. (2012). Development of a 3D Dynamic Programming Method for Weather Routing. Methods Algorithms Navig. Mar. Navig. Saf. Sea Transp..

[17] Wang H., Mao W., Eriksson L. (2019). A Three-Dimensional Dijkstra’s Algorithm for Multi-Objective Ship Voyage Optimization. Ocean Eng..

[18] Wang H., Lang X., Mao W. (2021). Voyage optimization combining genetic algorithm and dynamic programming for fuel/emissions reduction. Transp. Res. Part D.

[19] Mou J.M., van der Tak C., Ligteringen H. (2010). Study on collision avoidance in busy waterways by using AIS data. Ocean Eng..

[20] Fabbri T., Vicen-Bueno R. (2019). Weather-Routing System Based on METOC Navigation Risk Assessment. J. Mar. Sci. Eng..

[21] Karin T. (2020). Global Land Mask. https://zenodo.org/records/4066722.

[22] Zhang M., Tsoulakos N., Kujala P., Hirdaris S. (2024). Dataset Related to the Journal Article “A Deep Learning Method for the Prediction of Ship Fuel Consumption in Real Operational Conditions”. https://zenodo.org/records/13846854.

[23] MarineTraffic (2026). MarineTraffic: Global Ship Tracking Intelligence. https://www.marinetraffic.com/en/ais/home/centerx:-12.0/centery:25.0/zoom:4.

[24] National Geospatial-Intelligence Agency (NGA) (2024). World Port Index (WPI) Viewer. https://msi.nga.mil/Publications/WPI.

[25] Open-Meteo (2026). Marine Weather API Documentation. https://open-meteo.com/en/docs/marine-weather-api.

[26] International Maritime Organization (1972). Convention on the International Regulations for Preventing Collisions at Sea (COLREGs).

[27] European Marine Observation and Data Network (2026). EMODnet Bathymetry. https://www.nioz.nl/en/research/projects/emodnet-bathymetry.

[28] International Hydrographic Organization (2026). S-101 Electronic Navigational Chart Product Specification. https://iho.int/standards-and-specifications.

